# Risk factors associated with suicide among esophageal carcinoma patients from 1975 to 2016

**DOI:** 10.1038/s41598-021-98260-w

**Published:** 2021-09-21

**Authors:** Chongfa Chen, Huapeng Lin, Fengfeng Xu, Jianyong Liu, Qiucheng Cai, Fang Yang, Lizhi Lv, Yi Jiang

**Affiliations:** 1 Department of Hepatobiliary Surgery, Dongfang Hospital, Xiamen University, No.156, West Second Ring North Road, Gulou District, Fuzhou, People’s Republic of China; 2 Department of Intensive Care Unit, Affiliated Hangzhou First People’s Hospital, Zhejiang University School of Medicine, Hangzhou, People’s Republic of China; 3 Department of Cardiothoracic Surgery, 900 Hospital of the Joint Logistics Team, Fuzhou, People’s Republic of China; 4 Department of Hepatobiliary Surgery, 900 Hospital of the Joint Logistics Team, Fuzhou, People’s Republic of China

**Keywords:** Psychology, Cancer epidemiology

## Abstract

Throughout the world, esophageal cancer patients had a greater suicidal risk compared with ordinary people. Thus, we aimed to affirm suicide rates, standardized mortality rates, and underlying suicide-related risk factors of esophageal cancer patients. Patients suffering esophageal cancer were chosen from the Surveillance, Epidemiology, and End Results repository in 1975–2016. Suicide rates as well as standardized mortality rates in the patients were measured. Univariable and multivariable Cox regression had been adopted for establishing the latent suicide risk factors among patients suffering esophageal cancer. On multivariable Cox regression, gender (male *vs.* female, HR: 6.37), age of diagnosis (70–105 *vs*. 0–55, HR: 2.69), marital status, race (white race vs. black race, HR: 6.64; American Indian/Alaska Native, Asian/Pacific Islander *vs.* black race, HR: 8.60), histologic Grade (Grade III vs. Grade I, HR: 2.36), no surgery performed (no/unknown *vs.* yes, HR: 2.01), no chemotherapy performed were independent risk factors related to suicide in patients suffering esophageal cancer. Male sex, the older age, unmarried state, non-black race, histologic Grade III, no surgery performed, no chemotherapy performed were strongly related to suicide in patients suffering esophageal cancer.

## Introduction

Suicide has become a worldwide public health issue, or kind of sophisticated action subject to factors in physiology, psychology, society, environment and culture^1^. In addition, it is still the main contributor of death in people aged 15–24 globally, and also the tenth main cause of death across North America^2^. 817,000 people commit suicide worldwide in 2016, accounting for 1.49% in total deaths^3^. According to the World Health Organization (WHO), the 2016 suicide rate totaled 10.6 suicides per 100,000 persons, with 80% among middle-low income states^4^. Despite the decline of suicide by around 18% in 2000–2016 across most WHO areas^2^, the U.S. witnessed an annual increase of suicide by 1.5% after 2000^5^.

Recently, research has discovered depression is significantly correlated with suicide, and the suicide rate in depression patients far exceeds that in ordinary people^6–8^. During the COVID-19 outbreak and resulting quarantine, suicidal intention and action quickly increased in high-risk groups, including unemployed^9^, bereaved^10^, smoking^11^, alcohol consumption^12–14^, or even genetic level groups^15,16^. Although cancer patients of both genders underwent identical stress, drastic decline of family income possibly intensified the suicidal intention and action among men^17^. Much evidence has suggested a stronger propensity of desperation and suicide among patients with bad prognosis illnesses (in particular cancer)^18–21^. Moreover, many proofs in systematic reviews have revealed the growing suicidal risk in cancer patients^22–24^. It is surprising that suicide rate of U.S. cancer patients almost doubled that in ordinary people^25^. In addition, a latest research performed by Zaorsky et al. indicates standardized mortality rate (SMR) of suicide in cancer patients is 4.44 in comparison with ordinary people^26^. Given that suicide can be recognized and prevented, it is imperative to identify patients at high risk of suicide^27^.

Throughout the world, esophageal cancer has been considered the sixth most representative cancer-related death: 572,034 new cases and 508,585 deaths were discovered in 2018^28^. In 2019, Chelsea Anderson et al. found the SMR in esophageal cancer was 5.03 (95% Confidence Interval (CI): 4.03–6.19) in the U.S. general population (2000–2014), which might be adjusted by age, sex, as well as race^29^. Whereas, by far, only a limited number of reports have examined the suicide-related risk factors among esophageal cancer patients with a large sample size. Hence, the current study aims to measure suicide rates as well as SMRs in comparison with U.S. general population and recognize underlying factors associated with suicide by reference to the SEER database (1975–2016).

## Methods

### Data selection

Esophageal cancer patients, with diagnosis time in 1975—2016, had been chosen from the National Cancer Institute's Surveillance, Epidemiology, and End Results (SEER) Program. Data about the general U.S. population, demographic and clinical variables were gathered from the National Center for Health Statistics in 1975–2016 and acquired via the SEER Program^30,31^. Patients had been differentiated with primary site codes (C15.0-C15.5, C15.8, C15.9) related to esophageal cancer in line with International Classification of Diseases for Oncology codes (3rd edition) of esophageal cancer^32^. Main outcome was suicide-caused death, which might be recognized through the cause of death code (suicide or self-caused injury).

**Figure 1:**
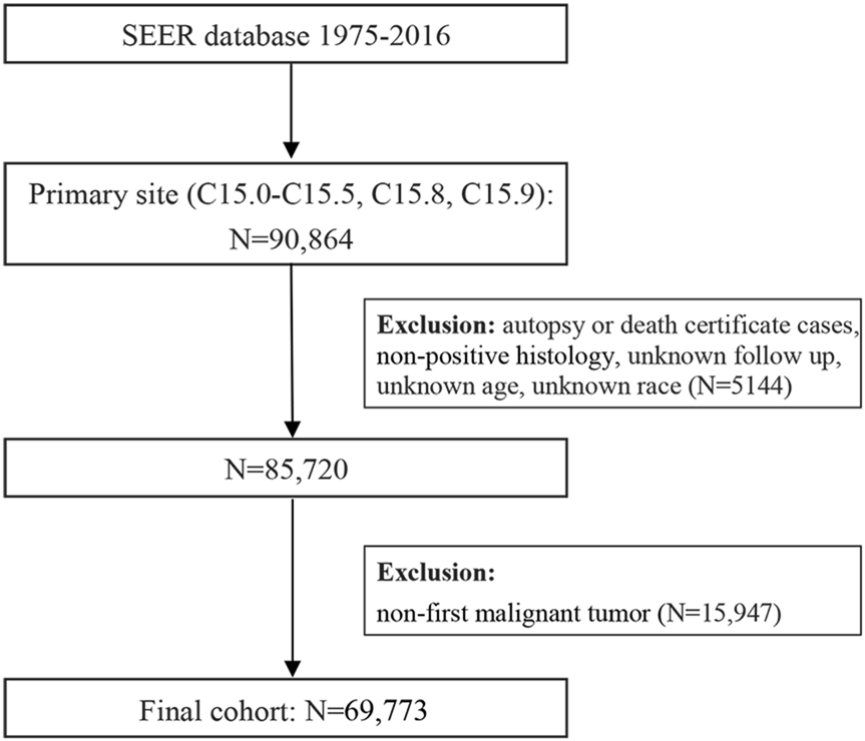
The flow diagram of patient selection (Description: There are steps of how to identify 161 suicidal patients from 90,864 esophageal cancer patients in Surveillance, Epidemiology, and End Results program during 1975–2016; SEER*Stat software, version 8.3.6, http://www.seer.cancer.gov/seerstat/; Microsoft Word software, version 16.0.14131.20296, https://www.microsoft.com/zh-cn/download/).

SEER*Stat software (version 8.3.6) was applied for establishing the patients^33^. Details are presented in Fig. 1.

### Statistical analysis

Suicide rates among patients suffering esophageal cancer were counted according to reported suicides per 100,000 person-years of follow-up. The U.S. population suicide rates at the National Center for Health Statistics were accessed from the SEER Program for a comparison with those of our cohort and ordinary people. Data were described using SMRs, which could be adjusted according to age, race, as well as sex in the U.S. population during the same period. Five-year age groupings were chosen in normalization^34^. SMRs were measured as the ratio of reported suicides in esophageal cancer patients to expected suicide counts of overall population. Expected suicide counts were calculated through multiplying overall population suicide rate by person-time of our cohort, considering the strata in age, race, and sex. Ninety-five percent of confidence interval (CI) in the SMRs was measured in the mid-*P* test^35^. In addition, between-group suicide rates were figured out by the chi-square test, and Bonferroni-corrected *P* value was used in multiple comparisons. Further, SMRs were evaluated in accordance with survival months (< 2 months, 2 months—11 months, 12 months—59 months, ≥ 60 months), and the initiative 2-month cutoff was chosen as the best estimation for the rational window between diagnosis and starting cancer therapy. The duration was supposed to be linked to the maximum suicide rate. To investigate interactions between different factors, we performed likelihood ratio testings to assess interactions among Sex, Age of diagnosis, SEER disease stage, Race, and Treatment performed (Surgery, Radiotherapy, and Chemotherapy). Univariable and multivariable Cox regression had been conducted to determine crude and adjusted hazard ratios (HRs) as well as 95% CI, to reveal underlying suicide-related risk factors. Merely variables satisfying *P* < 0.1 under univariate Cox regression model are proper for multivariate Cox regression model. In relevant analyses, patients who had 0-month follow-up were given a value of 0.5 months. Age of diagnosis was the sole continuous variable. For investigating suicide risk in patients at various age groups, X-tile software (http://tissuearray.org/) had been employed for discovering the optimal cutoffs of age (see Supplementary Fig. S1 online). Overall statistical analyses proved two-sided, and *P* < 0.05 demonstrated the statistical significance. SPSS (version 25.0, SPSS, Chicago, IL, USA), Microsoft Word (version 16.0.14131.20296) and Microsoft Excel (version 16.0.12730.20188, Microsoft, Redmond, State of Washington) were adopted for carrying out the statistical analyses.

### Ethic declarations

The research involved no human participants or infringement of individual privacy. Thus, approval from the institutional review board was unnecessary. Informed consent was abandoned in the anonymous study.

## Results

### Patient baseline features

In general, 69,773 esophageal cancer patients had been determined from the SEER repository in 1975–2016, encompassing 53,665 males and 16,108 females. Of which, 161 of them (0.23%) commit suicide, 60,113 of them (86.16%) died of other reasons, whereas 9499 patients (13.61%) were alive (Table 1). The steps of choosing patients were depicted in Fig. 1. Among all patients, 38,027 (54.50%) patients had got married or mates, whereas 17,819 (25.54%) patients had once got married (divorced, widowed and separated), and 10,690 (15.32%) of them were single (never married). White race (80.73%) was the predominant race. Overall, 19,228 (27.56%) of them received cancer-directed surgery, whereas 37,400 (53.60%) patients receive chemotherapy. Regarding the patients who committed suicide, 152 (94.41%) were males, and 9 (5.59%) females. For marital status, 81 (50.31%) had got married or mates (domestic partners), whereas 41 (25.47%) were previously married, and 28 (17.39%) were single. Likewise, white (90.68%) was also a prominent race. 47 (29.19%) patients underwent cancer-directed surgery, while merely 79 (49.07%) patients had chemotherapy. Table 1 listed patient demographics as well as clinical characteristics.

**Table 1:** Baseline characteristics of patients with esophageal cancer stratified by suicidal death, nonsuicidal death and alive patients.

Variables	Overall	Suicidal death	Nonsuicidal death	Alive Patients
	N (%)	N (%)	N (%)	N (%)
Patients	69,773 (100%)	161 (100%)	60,113 (100%)	9499 (100%)
**Year of diagnosis**				
1975–1988	9139 (13%)	22 (14%)	9076 (15%)	41 (0%)
1989–2002	19,512 (28%)	51 (32%)	18,664 (31%)	797 (8%)
2003–2016	41,122 (59%)	88 (55%)	32,373 (54%)	8661 (91%)
**Sex**				
Male	53,665 (77%)	152 (94%)	46,111 (77%)	7402 (78%)
Female	16,108 (23%)	9 (6%)	14,002 (23%)	2097 (22%)
**Age at diagnosis**				
0–55	12,994 (19%)	18 (11%)	10,787 (18%)	2189 (23%)
56–69	29,437 (42%)	63 (39%)	24,727 (41%)	4647 (49%)
70–105	27,342 (39%)	80 (50%)	24,599 (41%)	2663 (28%)
**Marital status**				
Married/ Domestic Partner	38,027 (55%)	81 (50%)	32,156 (53%)	5790 (61%)
Previously Married^a^	17,819 (26%)	41 (25%)	16,115 (27%)	1663 (18%)
Single^b^	10,690 (15%)	28 (17%)	9185 (15%)	1477 (16%)
Unknown	3237 (5%)	11 (7%)	2657 (4%)	569 (6%)
**Race**				
White	56,327 (81%)	146 (91%)	48,011 (80%)	8170 (86%)
Black	9606 (14%)	3 (2%)	8898 (15%)	705 (7%)
American Indian/Alaska Native, Asian/Pacific Islander	3840 (6%)	12 (7%)	3204 (5%)	624 (7%)
**Histologic grade**				
Grade I	3621 (5%)	6 (4%)	2872 (5%)	743 (8%)
Grade II	22,519 (32%)	44 (27%)	19,027 (32%)	3448 (36%)
Grade III	28,516 (41%)	79 (49%)	25,343 (42%)	3094 (33%)
Grade IV	1531 (2%)	4 (2%)	1409 (2%)	118 (1%)
Unknown	13,586 (19%)	28 (17%)	11,462 (19%)	2096 (22%)
**Primary site**				
Lower third of esophagus	38,000 (54%)	105 (65%)	31,653 (53%)	6242 (66%)
Middle third of esophagus	13,460 (19%)	19 (12%)	12,167 (20%)	1274 (13%)
Upper third of esophagus	3967 (6%)	8 (5%)	3546 (6%)	413 (4%)
Overlapping lesion of esophagus	3351 (5%)	10 (6%)	3023 (5%)	318 (3%)
Cervical esophagus	1597 (2%)	5 (3%)	1414 (2%)	178 (2%)
Thoracic esophagus	2281 (3%)	4 (2%)	2006 (3%)	271 (3%)
Abdominal esophagus	672 (1%)	0 (0%)	591 (1%)	81 (1%)
Esophagus, NOS	6445 (9%)	10 (6%)	5713 (10%)	722 (8%)
**Histology recode—broad groupings**				
Adenomas and adenocarcinomas	33,797 (48%)	88 (55%)	27,610 (46%)	6099 (64%)
Squamous cell neoplasms	28,892 (41%)	57 (35%)	26,230 (44%)	2605 (27%)
Others	7084 (10%)	16 (10%)	6273 (10%)	795 (8%)
**SEER disease stage**				
Localized	15,873 (23%)	43 (27%)	12,341 (21%)	3489 (37%)
Regional	20,978 (30%)	55 (34%)	17,153 (29%)	3770 (40%)
Distant	23,622 (34%)	40 (25%)	22,014 (37%)	1568 (17%)
Unknown/unstaged	9300 (13%)	23 (14%)	8605 (14%)	672 (7%)
**Surgery performed**				
Yes	19,228 (28%)	47 (29%)	13,919 (23%)	5262 (55%)
No/unknown	50,545 (72%)	114 (71%)	46,194 (77%)	4237 (45%)
**Radiotherapy performed**				
Yes	39,423 (57%)	88 (55%)	33,735 (56%)	5600 (59%)
No/unknown	30,350 (43%)	73 (45%)	26,378 (44%)	3899 (41%)
**Chemotherapy performed**				
Yes	37,400 (54%)	79 (49%)	31,197 (52%)	6124 (64%)
No/unknown	32,373 (46%)	82 (51%)	28,916 (48%)	3375 (36%)

SEER, the surveillance, epidemiology, and end results program.

^a^Included divorced, widowed and separated.

^b^Included never married.

### Difference in suicide rates and SMRs

#### Suicide rates

During 1975 and 2016, 161 suicide cases had been reported in 69,773 esophageal cancer patients surveyed for 128,508.08 person-years, resulting in the suicide rate of 125.28 per 100,000 person-years. Higher suicide rates in esophageal cancer patients correlated with male sex (*vs.* female sex, *P* < 0.001), white race (*vs.* black race, *P* < 0.001), as well as the middle third of the esophagus (*vs.* lower third of the esophagus, *P* < 0.01). The chi-square test of linear trend revealed growing suicide rate in esophageal cancer patients with age of diagnosis (*P* < 0.01) as well as survival months (*P* < 0.01). However, there were no significant discrepancies about suicide rates concerning year of diagnosis, marital status, histology recode-broad groupings, histologic grade, SEER disease stage, surgical procedures performed, radiotherapy performed, and chemotherapeutic options administered. Details are presented in Table 2.

**Table 2:** Suicide rates and SMRs among patients with esophageal cancer by demographic and clinic characteristics (1975‐2016).

Variables	Suicidal death	Person‐years	Suicide rate per 100,000 person‐years	*P*	SMR^a^	95% CI	
						Lower	Upper
Total	161	128,508.08	125.28		5.45	4.66	6.35
**Year of diagnosis**							
1975–1988	22	13,465.71	163.38	0.389^$^	8.37	5.38	12.46
1989–2002	51	43,893.58	116.19		5.23	3.94	6.82
2003–2016	88	71,148.79	123.68		5.13	4.14	6.29
**Sex**							
Male	152	98,368.04	154.52	** < 0.001**	12.72	10.81	14.86
Female	9	30,140.04	29.86	Ref	2.47	1.20	4.53
**Age at diagnosis**							
0–55	18	30,783.29	58.47	** < 0.01**^$^	2.68	1.64	4.15
56–69	63	59,465.21	105.94		5.04	3.91	6.41
70–105	80	38,259.58	209.10		7.76	6.20	9.61
**Marital status**							
Married/domestic partner	81	80,203.63	100.99	Ref	4.03	3.22	4.99
Previously Married^b^	41	25,983.88	157.79	0.687^#^	8.67	6.30	11.64
Single^c^	28	16,431.25	170.41		8.40	5.69	11.98
Unknown	11	5,889.33	186.78		7.96	4.19	13.84
**Race**							
Black	3	13,759.79	21.80	Ref	1.43	0.36	3.89
White	146	107,948.29	135.25	** < 0.001**^#^	8.10	6.86	9.49
American Indian/Alaska Native, Asian/Pacific Islander	12	6,800.00	176.47		11.24	6.09	19.11
**Primary site**							
Lower third of esophagus	105	76,536.13	137.19	Ref	5.42	4.45	6.53
Middle third of esophagus	19	21,667.38	87.69	** < 0.01**^#^	4.91	3.05	7.53
Upper third of esophagus	8	6,392.58	125.15		6.59	3.06	12.51
Overlapping lesion of esophagus	10	4,514.21	221.52		10.33	5.25	18.42
Cervical esophagus	5	3,228.25	154.88		8.59	3.15	19.04
Thoracic esophagus	4	3,828.54	104.48		5.67	1.80	13.67
Abdominal esophagus	0	1,539.79	0		0	-	-
Esophagus, NOS	10	10,801.21	92.58		4.13	2.10	7.36
**Histology recode—broad groupings**							
Adenomas and adenocarcinomas	88	68,622.50	128.24	Ref	4.75	3.83	5.82
Squamous cell neoplasms	57	47,955.46	118.86	0.101^#^	7.01	5.36	9.02
Others	16	11,930.13	134.11		5.59	3.31	8.89
**Histologic grade**							
Grade I	6	9,886.42	60.69	0.660^$^	2.60	1.05	5.40
Grade II	44	45,318.71	97.09		4.31	3.17	5.73
Grade III	79	44,038.88	179.39		7.66	6.11	9.50
Grade IV	4	2,386.58	167.60		7.45	2.37	17.97
Unknown	28	26,877.50	104.18		4.55	3.08	6.49
**SEER disease stage**							
Localized	43	51,467.67	83.55	0.151^$^	3.58	2.62	4.77
Regional	55	43,632.46	126.05		5.48	4.17	7.08
Distant	40	19,579.63	204.29		8.92	6.46	12.02
Unknown/unstaged	23	13,828.33	166.33		7.73	5.02	11.42
**Surgery performed**							
Yes	47	68,214.04	68.90	Ref	2.90	2.16	3.82
No/unknown	114	60,294.04	189.07	0.642	8.56	7.09	10.25
**Radiotherapy performed**							
Yes	88	75,473.25	116.60	Ref	5.23	4.22	6.41
No/unknown	73	53,034.83	137.65	0.637	5.75	4.54	7.19
**Chemotherapy performed**							
Yes	79	75,153.46	105.12	Ref	4.65	3.71	5.76
No/unknown	82	53,354.63	153.69	0.248	6.54	5.24	8.08
**Survival months**							
< 2 months	35	670.50	5,219.99	** < 0.01**^$^	216.79	153.36	298.17
2 months-11 months	74	14,686.83	503.85		21.57	17.05	26.92
12 months-59 months	41	44,284.42	92.58		3.89	2.83	5.23
> = 60 months	11	68,866.33	15.97		0.71	0.38	1.24

SMR, standardized mortality ratio; SEER, the surveillance, epidemiology, and end results program; 95% CI, 95% confidence interval; NOS, Not Otherwise Specified.

^a^SMR was adjusted by age, race, and sex to the US population over the same time. Five-year age categories were used for standardization using SEER*Stat 8.3.6 and Microsoft Excel 16.0.12730.20188 (Microsoft, Redmond, Washington).

^b^Included divorced, widowed and separated.

^c^Included never married.

^#^The Bonferroni-corrected *P* value was used for multiple comparisons.

^$^The chi-square test for linear trend was used for ordinal multi-categorical variables.

The *P* values in the bold are statistically significant.

#### SMRs

SMRs were used for a comparison on suicide fatality rate between studied population and general population. An SMR as 5.45 (95% CI: 4.66–6.35) was reported between esophageal cancer patients and U.S. general population, with 12.72 (95% CI: 10.81–14.86) for males, 2.47 (95% CI: 1.20–4.53) for females, 8.10 (95% CI: 6.86–9.49) in white race, 1.43 (95% CI: 0.36–3.89) in black race, and 11.24 (95% CI: 6.09–19.11) in other races (American Indian/Alaska Native, Asian/Pacific Islander). Suicide rates generally declined from 1975 to 2016 (1975–1988, SMR: 8.37, CI: 5.38–12.46; 1989–2002, SMR: 5.23, CI: 3.94–6.82; 2003–2016, SMR: 5.13, CI: 4.14–6.29), regardless of the lack of any statistical pattern (*P* = 0.389). Remarkably elevated suicide rates in esophageal cancer patients were observed during the first five years after cancer diagnosis (< 2 months, SMR: 216.79, 95% CI: 153.36–298.17; 2 months-11 months, SMR: 21.57, 95% CI: 17.05–26.92; 12 months-59 months, SMR: 3.89, 95% CI: 2.83–5.23, *P* < 0.01). Details are presented in Table 2.

### Risk factors

After multiple testing, no statistically significant interactions were observed among these risk factors. Details are presented as Supplementary Table S1 online. Univariable Cox regression findings confirmed a significant correlation with high suicide risk based on gender (male *vs*. female, HR: 5.04, 95% CI: 2.57–9.86, *P* < 0.001), age of diagnosis (70–105 *vs*. 0–55, HR: 2.81, 95% CI: 1.68–4.70, *P* < 0.001), race (white race *vs*. black, HR: 7.03, 95% CI: 2.24–22.06, *P* < 0.001; American Indian/Alaska Native, Asian/Pacific Islander *vs.* black race, HR: 8.91, 95% CI: 2.51–31.56, *P* < 0.001), histologic grade (grade III *vs.* grade I, HR: 2.30, 95% CI: 1.00–5.27, *P* = 0.050), surgery performed (no/unknown *vs.* yes, HR: 1.82, 95% CI: 1.28–2.58, *P* < 0.001), chemotherapy performed (no/unknown *vs.* yes, HR: 1.57, 95% CI: 1.15–2.14, *P* < 0.01) (Table 3). Multivariable Cox regression outcomes showed gender (male *vs.* female, HR: 6.37, 95% CI: 3.21–12.67, *P* < 0.001), age of diagnosis (70–105 *vs*. 0–55, HR: 2.69, 95% CI: 1.58–4.57, *P* < 0.001), marital status (previously married *vs.* married/mate, HR: 1.75, 95% CI: 1.19–2.57, *P* < 0.01; bachelor (single) *vs.* married/mate, HR: 2.07, 95% CI: 1.33–3.21, *P* < 0.01), race (white race *vs.* black race, HR: 6.64, 95% CI: 2.10–21.06, *P* < 0.01; American Indian/Alaska Native, Asian/Pacific Islander vs. black race, HR: 8.60, 95% CI: 2.41–30.66, *P* < 0.001), histologic grade (grade III vs. grade I, HR: 2.36, 95% CI: 1.03–5.45, *P* = 0.044), surgery performed (no/unknown vs. yes, HR: 2.01, 95% CI: 1.38–2.93, *P* < 0.001), chemotherapy performed (no/unknown vs. yes, HR: 1.72, 95% CI: 1.18–2.49, *P* < 0.01) might predict suicide. Table 3 described all the details linked to suicide indexes of the whole cohort.

**Table 3:** Univariable and multivariable analysis for the suicide of esophageal cancer patients.

Variables	Univariable analysis				Multivariable analysis			
	HR	95% CI		*P*	HR	95% CI		*P*
		Lower	Upper			Lower	Upper	
**Year of diagnosis**								
1975–1988	1.32	0.83	2.11	0.247				
1989–2002	1.14	0.81	1.62	0.453				
2003–2016	Ref			0.464				
**Sex**								
Male	5.04	2.57	9.86	** < 0.001**	6.37	3.21	12.67	** < 0.001**
Female	Ref				Ref			
**Age at diagnosis**								
0–55	Ref			** < 0.001**	Ref			** < 0.001**
56–69	1.66	0.98	2.80	**0.059**	1.70	1.00	2.88	0.051
70–105	2.81	1.68	4.70	** < 0.001**	2.69	1.58	4.57	** < 0.001**
**Marital status**								
Married/Domestic Partner	Ref			0.109	Ref			** < 0.01**
Previously Married^a^	1.34	0.92	1.95	0.132	1.75	1.19	2.57	** < 0.01**
Single^b^	1.49	0.97	2.29	**0.070**	2.07	1.33	3.21	** < 0.01**
Unknown	1.76	0.94	3.31	**0.078**	1.83	0.96	3.46	0.065
**Race**								
Black	Ref			** < 0.01**	Ref			** < 0.01**
White	7.03	2.24	22.06	** < 0.001**	6.64	2.10	21.06	** < 0.01**
American Indian/Alaska Native, Asian/Pacific Islander	8.91	2.51	31.56	** < 0.001**	8.60	2.41	30.66	** < 0.001**
**Primary site**								
Lower third of esophagus	Ref			0.318				0.430
Middle third of esophagus	0.58	0.36	0.95	**0.029**	0.68	0.41	1.12	0.131
Upper third of esophagus	0.83	0.40	1.70	0.612	0.83	0.40	1.73	0.621
Overlapping lesion of esophagus	1.38	0.72	2.64	0.330	1.42	0.74	2.72	0.295
Cervical esophagus	1.15	0.47	2.81	0.767	1.28	0.52	3.18	0.597
Thoracic esophagus	0.70	0.26	1.89	0.480	0.80	0.29	2.17	0.656
Abdominal esophagus	0	0	1.69E + 94	0.930	0	0	8.99E + 96	0.932
Esophagus, NOS	0.66	0.35	1.26	0.210	0.58	0.30	1.12	0.103
**Histology recode—broad groupings**								
Adenomas and adenocarcinomas	Ref			0.681				
Squamous cell neoplasms	0.87	0.62	1.21	0.395				
Others	1.00	0.59	1.70	0.998				
**Histologic grade**								
Grade I	Ref			**0.030**				**0.025**
Grade II	1.38	0.59	3.25	0.455	1.46	0.62	3.43	0.390
Grade III	2.30	1.00	5.27	**0.050**	2.36	1.03	5.45	**0.044**
Grade IV	2.22	0.63	7.87	0.217	2.31	0.65	8.22	0.195
Unknown	1.51	0.63	3.66	0.357	1.44	0.59	3.49	0.421
**SEER disease stage**								
Localized	Ref			0.440				
Regional	1.21	0.81	1.81	0.357				
Distant	1.31	0.84	2.05	0.233				
Unknown/unstaged	1.49	0.89	2.48	0.126				
**Surgery performed**								
Yes	Ref				Ref			
No/unknown	1.82	1.28	2.58	** < 0.001**	2.01	1.38	2.93	** < 0.001**
**Radiotherapy performed**								
Yes	Ref				Ref			
No/unknown	1.30	0.95	1.78	**0.096**	1.06	0.73	1.54	0.761
**Chemotherapy performed**								
Yes	Ref				Ref			
No/unknown	1.57	1.15	2.14	** < 0.01**	1.72	1.18	2.49	** < 0.01**

SMR, standardized mortality ratio; SEER, the surveillance, epidemiology, and end results; HR, Hazard Ratio; 95% CI, 95% confidence interval; NOS, Not Otherwise Specified.

^a^Included divorced, widowed and separated.

^b^Included never married.

^#^The Bonferroni-corrected *P* value was used for multiple comparisons.

^$^The chi-square test for linear trend was used for ordinal multi-categorical variables.

The *P* and HR values in the bold were statistically significant or considered to be analyzed in multivariate regression model.

## Discussion

By reference to associated surveys, suicide risk in cancer patients across various countries has gone up^24,25,36,37^. To be specific, the Italian data analysis performed by Ravaioli. A. et al. verified the growing suicide risk among cancer patients (pooled SMR: 1.7; 95% CI: 1.5–1.9)^24^. In addition to the finding, scholars in Norway (HR: 2.5; 95% CI: 1.7–3.8)^38^, Lithuania (SMR:1.62; 95% CI: 1.27–2.06)^39^, the U.K. (SMR: 1.20, 95% CI: 1.16–1.25)^36^, as well as the U.S. (SMR: 2.06; 95% CI: 2.00–2.12) have also given alike reports over the past few decades^25^. A novel contribution of this research is that analysis on suicide-associated risk factors among esophageal cancer patients on the basis of SEER database, which has the largest sample size at present, provides an important basis for clinical prevention and intervention of esophageal cancer suicide. As indicated by the population-based research, suicide rate among esophageal cancer patients reached up to 125.28 per 100,000 person-years, while gross SMR amounted to 5.45 (95% CI: 4.66–6.35). Male sex (SMR: 12.72), diagnosed at an older age (SMR: 7.76), unmarried state, non-black race, histologic grade III (SMR: 7.66), no surgery performed (SMR: 8.56) and no chemotherapy performed (SMR: 6.54) might significantly increase suicide rate in esophageal cancer patients. Details are presented in Table 2.

The SMR results of the above risk factors suggested suicide rates among patients suffering esophageal cancer were obviously greater compared with those of the general U.S. population, especially in men, older age, patients without chemotherapy or surgery performed. According to our results in multivariate analysis, the suicide rates among patients suffering esophageal cancer were subject to multiple demographic features, histopathologic characteristics, as well as treatment therapies. Whereas, corresponding risk factors related to suicide among patients suffering esophageal cancer varied from those of the non-cancer population in the United States^40–42^. Therefore, it was necessary to be complemented with relevant references and present the links between suicide and cancer (e.g., common risk factors such as alcohol^12–14^, smoking^11^, risk behaviours^43–45^, genetics^15,16^, and increased stress due to cancer diagnosis^26,37,46,47^). Additional points to the relationship between cancer and suicide, we have elaborated on reasons for not proceeding to treatment (chemotherapy, surgery).

### Gender

In Tables 2, 3, the male suicide rate (154.52 per 100,000 person-years) was almost five times larger relative to the female suicide rate (*P* < 0.001). Besides that, males had a higher risk of committing suicide in contrast to females, with an HR of 6.34 in our results, which was corresponding to some previous findings, such as those for the general population^48^, as well as patients suffering other cancer diseases, like lung cancer (SMR in males: 4.61, 95% CI: 4.34–4.90; SMR in females: 3.02, 95% CI: 2.53–3.58)^49^, gastric carcinomas (SMR in males: 4.85, 95% CI: 3.89–5.98; SMR in females: 3.74, 95% CI: 1.94–6.48)^25,26^. Although cancer patients of both genders possibly had experienced the same stress^50,51^, smoking^11^, alcohol consumption^12–14^, dramatic decline of family income urged men to generate growing suicidal intention and action^17^.

### Age at diagnosis

The current research reported a significant growing trend of suicide rate in the elderly (70–105 *vs.* 0–55, HR: 2.69, 95% CI: 1.58–4.57, *P* < 0.001), as shown by Tables 2, 3. Recently, a few studies have further defined older age as a suicide-related risk factor in patients suffering from cancer diseases as well as ordinary people^37,52^. However, exceptions did exist. According to the research carried out by Gaitanidis A et al. and Kroenke CH et al., patients at a younger age had a greater potential of committing suicide in comparison with elder breast cancer patients^53,54^. A possible reason was young females showed a stronger propensity for desperation in physiology and psychology compared with middle-aged and elder counterparts following breast cancer diagnosis. This intensified their suicidal action and intention^53^.

### Marital status

The current study found that unmarried state (previously married *vs.* married/domestic partner, HR: 1.75, 95% CI: 1.19–2.57, *P* < 0.01, single *vs.* married/domestic partner, HR: 2.07, 95% CI: 1.33–3.21, *P* < 0.01) was not protective against suicide in individuals with esophageal cancer. Additionally, a total of 36,221 patients with pancreatic adenocarcinoma was analyzed by Kiran K. Turaga al et., and its results also showed that the SMR of single and married was respectively 16.3 (95% CI: 14.3–18.6) and 6.4 (95% CI: 5.2–7.8), comparing to U.S. population aged 65 to 74 years old^55^. Besides, this trend was also consistent with patients suffering kidney cancers^56^, head and neck cancers as well as genitourinary malignancies^57,58^, which might be attributed to the superior physical quality, higher socioeconomic rank, and greater emotional support and social attention of the married^43–45^.

### Race

In addition, the research continued to inspect all risk factors related to suicide of patients from the perspective of race. Research results found that the white race proved to be one risk factor, which contributed to suicide, and the suicide rate of white (vs. black race, HR: 6.64, 95% CI: 2.10–21.06, P < 0.01) was 135.25 per 100,000 person-years. The finding demonstrated that the white race was possibly a major predictor related to suicide among cancer patients. Further, white race is considered as another risk factor related to suicide in a good number of studies^54,59^. As to the low suicide rate in black race, the most plausible reason can be probably attributable to the influence of genetics^15,16^, religious beliefs, family support as well as suicide-rejection culture^60–62^.

### Histologic grade

Regarding distinct clinical variables of esophageal cancer in Table 3, the patients with higher histologic grade (Grade III *vs.* Grade I, HR: 2.36, 95% CI: 1.03–5.45, *P* = 0.044) were considered to be at a higher suicide risk in contrast to those of lower histologic grade. It was universally recognized that low histologic grade represented cancer cells with well differentiation, denoting favorable prognosis and improved living standards^63^.

### Treatment performed

As depicted by Table 3, a factor linked to suicide was no cancer-directed surgery conducted (HR: 2.01, 95% CI: 1.38–2.93, P < 0.001), which implied that the possibility of suicide in esophageal cancer patients with surgical indications might also be a factor that should not be ignored. Likewise, Anderson. C. et al. claimed patients suffering from cancer diseases linked to the digestive system who received surgery had a lower propensity of committing suicide, compared with those not undergoing surgical treatment (SMR: 5.20, 95% CI: 4.64–5.81)^29^. Besides, the results from Samawi, H. H. et al. and also proved that no surgery was an independent risk factor^64^. We further found that no chemotherapy performed (HR: 1.72, 95% CI: 1.18–2.49, *P* < 0.01) predicted higher suicide risks compared with those with chemotherapies. Fortunately, the combination chemotherapy regimen was still one of the main treatments for esophageal cancer, particularly among patients with advanced or metastatic tumors.

Findings obtained in the current research basically conformed to a former survey which discovered that maximal standardized mortality ratios (SMRs) of suicide of cancer patients could be seen from those who had more serious tumour grades as well as those who had not received therapy^49^. Patients having advanced cancers were likely to experience more sufferings and showed a stronger propensity to anxiety or depression in comparison with those having early tumours^65^. A reasonable explanation for the correlation of therapy with lower suicide risk was that post-cancer diagnosis treatment provided more comfort and further reinforced their confidence in rehabilitation. This, to some extent, relieved the suffering caused by cancers^66^. For radiotherapy, we speculate that it may be because the dysphagia of patients after radiotherapy would not improve immediately. In most cases, patients would have weakness, neck and shoulder pain, and other symptoms after radiotherapy, which may increase the pressure and discomfort of patients. However, the tumor size after radiotherapy may be further reduced, increasing the resectability rate of surgery, and the prognosis may be improved, thus alleviating the pessimistic mood of patients^67^.

Therefore, the effect of radiotherapy on suicide in patients with esophageal cancer may not be significant, but the role of chemotherapy and surgery on suicide prevention can not be underestimated.

### Survival months

Survival months proved to be a main suicide risk factor in esophageal cancer patients, in particular two months following diagnosis (SMR: 216.79, 95% CI: 153.36–298.17; Table 2). In good agreement with former studies for other cancers, suicide risk among esophageal cancer patients often seemed better in the early stage following diagnosis compared with that in other stages, underscoring the necessity for social support and monitoring of esophageal cancer patients during such particular periods^26,37,46,47^. The government, clinicians, as well as family members are supposed to make regular evaluations on esophageal cancer patients about their suicide attempts or potential suicide risk actions, and meanwhile, use proper strategies to lower their suicide risk, particularly among patients diagnosed within two months^26,68^.

Additionally, examining variables not included by the SEER dataset, in particular those about perceived discrimination, as well as sentiment of estrangement from the mainstream culture seems necessary.

## Limitations

There are many inevitable constraints in the current study, such as rich retrospective data in SEER. Underlying confounders, including comorbidities, cancer recurrences, socioeconomic status, health insurance, underlying psychiatric diseases, suicide attempts, as well as details about therapeutic interventions cannot be used for further analysis because of the non-availability of corresponding data sources in the SEER program. However, by far, it remains to be the most all-round investigation about the subject. Moreover, incomplete information on psychological status is a common issue among patients with physical illness (i.e., cancer). Further work should be conducted to improve the prediction ability by applying more appropriate models incorporating other potential risk factors. Due to the retrospective design of this study, it was difficult to explain some ratings. Besides, anonymization of information inhibited the verification of whether respondent descriptions had precisely figured out the events happened^69^.

## Conclusions

To sum up, males, with older ages (70–105), bachelor, non-black race, histologic grade III, no surgical treatment or chemotherapy performed constituted remarkable indicators of suicide among esophageal cancer.

## Supplementary Information

Supplementary Information.

## Data Availability

Data involved in the research can be provided by the corresponding author if required.
