# Supplementary material for: Risk factors associated with suicide among esophageal carcinoma patients from 1975 to 2016
Source: Sci Rep. 2021 Sep 21;11:18766. doi: 10.1038/s41598-021-98260-w (PMC8455550; doi:10.1038/s41598-021-98260-w)
Supplement: Supplementary file 1 — Supplementary Information. [file 41598_2021_98260_MOESM1_ESM.docx]

**Supplementary Figure S1**


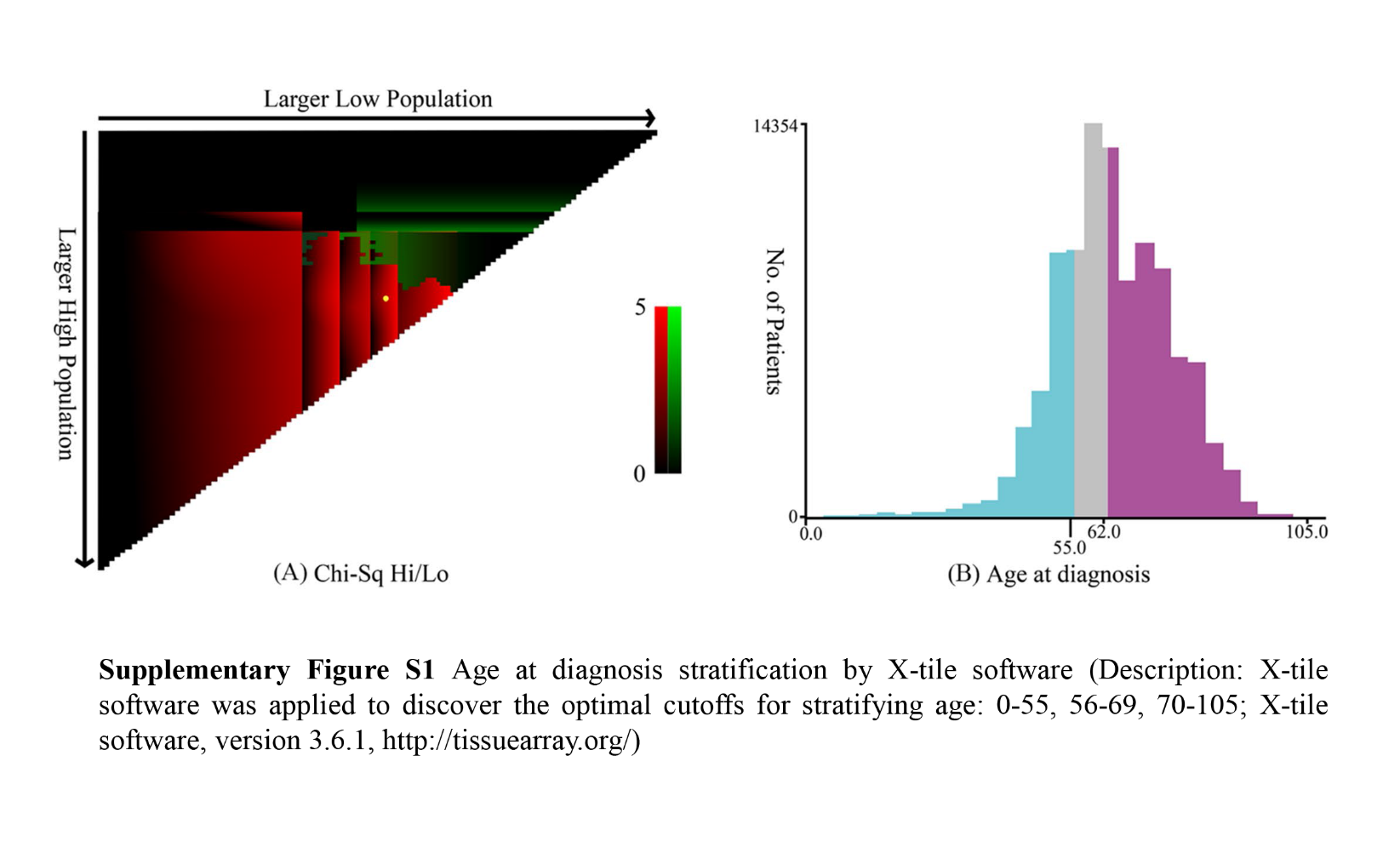


| **Supplementary Table S1** Interaction tests among Sex, Age of diagnosis, Race, SEER disease stage and Treatment performed | | | | | | | |
| --- | --- | --- | --- | --- | --- | --- | --- |
| Effect modifier | Exposure factor | N | HR | 95% CI | | P value | P interaction^a^ |
|  |  |  |  | Lower | Upper |  |  |
| **Age of diagnosis** | **Sex** |  |  |  |  |  | 0.349 |
| 11-55 | Male | 10708 | Ref |  |  |  |  |
| 11-55 | Female | 2286 | 0.57 | 0.131 | 2.483 | 0.455 |  |
| 56-69 | Male | 23811 | 1.87 | 1.076 | 3.246 | 0.027 |  |
| 56-69 | Female | 5626 | 0.24 | 0.055 | 1.039 | 0.056 |  |
| 70-105 | Male | 19146 | 3.48 | 2.018 | 5.993 | <0.001 |  |
| 70-105 | Female | 8196 | 0.55 | 0.201 | 1.502 | 0.243 |  |
| Total | Male | 53665 | Ref |  |  |  |  |
| Total | Female | 16108 | 0.18 | 0.091 | 0.348 | <0.001 |  |
| **Race** | **Sex** |  |  |  |  |  | 0.340 |
| Black | Male | 6798 | Ref |  |  |  |  |
| Black | Female | 2808 | 0.00 | 0 | Inf | 0.991 |  |
| White | Male | 43893 | 5.88 | 1.873 | 18.461 | 0.002 |  |
| White | Female | 12434 | 1.07 | 0.277 | 4.143 | 0.921 |  |
| Other | Male | 2974 | 6.79 | 1.869 | 24.683 | 0.004 |  |
| Other | Female | 866 | 4.12 | 0.688 | 24.658 | 0.121 |  |
| Total | Male | 53665 | Ref |  |  |  |  |
| Total | Female | 16108 | 0.21 | 0.107 | 0.409 | <0.001 |  |
| **SEER disease stage** | **Sex** |  |  |  |  |  | 0.774 |
| Localized | Male | 11735 | Ref |  |  |  |  |
| Localized | Female | 4138 | 0.15 | 0.037 | 0.624 | 0.009 |  |
| Regional | Male | 16362 | 1.13 | 0.748 | 1.714 | 0.556 |  |
| Regional | Female | 4616 | 0.32 | 0.116 | 0.905 | 0.032 |  |
| Distant | Male | 19165 | 1.23 | 0.775 | 1.935 | 0.384 |  |
| Distant | Female | 4457 | 0.27 | 0.065 | 1.118 | 0.071 |  |
| Unknown/unstaged | Male | 6403 | 1.63 | 0.964 | 2.74 | 0.068 |  |
| Unknown/unstaged | Female | 2897 | 0.17 | 0.023 | 1.227 | 0.079 |  |
| Total | Male | 53665 | Ref |  |  |  |  |
| Total | Female | 16108 | 0.20 | 0.1 | 0.385 | <0.001 |  |
| **Surgery performed** | **Sex** |  |  |  |  |  | 0.978 |
| Yes | Male | 15607 | Ref |  |  |  |  |
| Yes | Female | 3621 | 0.19 | 0.046 | 0.782 | 0.021 |  |
| No/Unknown | Male | 38058 | 1.97 | 1.375 | 2.832 | <0.001 |  |
| No/Unknown | Female | 12487 | 0.37 | 0.164 | 0.815 | 0.014 |  |
| Total | Male | 53665 | Ref |  |  |  |  |
| Total | Female | 16108 | 0.19 | 0.095 | 0.366 | <0.001 |  |
| **Radiotherapy performed** | **Sex** |  |  |  |  |  | 0.426 |
| Yes | Male | 30409 | Ref |  |  |  |  |
| Yes | Female | 9014 | 0.15 | 0.056 | 0.417 | <0.001 |  |
| No/Unknown | Male | 23256 | 1.24 | 0.898 | 1.706 | 0.193 |  |
| No/Unknown | Female | 7094 | 0.33 | 0.133 | 0.808 | 0.015 |  |
| Total | Male | 53665 | Ref |  |  |  |  |
| Total | Female | 16108 | 0.20 | 0.102 | 0.392 | <0.001 |  |
| **Chemotherapy performed** | **Sex** |  |  |  |  |  | 0.491 |
| Yes | Male | 29831 | Ref |  |  |  |  |
| Yes | Female | 7569 | 0.14 | 0.045 | 0.457 | 0.001 |  |
| No/Unknown | Male | 23834 | 1.59 | 1.152 | 2.184 | 0.005 |  |
| No/Unknown | Female | 8539 | 0.37 | 0.162 | 0.857 | 0.020 |  |
| Total | Male | 53665 | Ref |  |  |  |  |
| Total | Female | 16108 | 0.19 | 0.099 | 0.38 | <0.001 |  |
| **Race** | **Age of diagnosis** |  |  |  |  |  | 0.639 |
| Black | 11-55 | 2686 | Ref |  |  |  |  |
| Black | 56-69 | 4531 | 1084827.66 | 0 | Inf | 0.992 |  |
| Black | 70-105 | 2389 | 4693216.50 | 0 | Inf | 0.991 |  |
| White | 11-55 | 9645 | 5954162.01 | 0 | Inf | 0.991 |  |
| White | 56-69 | 23334 | 9547550.00 | 0 | Inf | 0.990 |  |
| White | 70-105 | 23348 | Inf | 0 | Inf | 0.990 |  |
| Other | 11-55 | 663 | Inf | 0 | Inf | 0.990 |  |
| Other | 56-69 | 1572 | Inf | 0 | Inf | 0.990 |  |
| Other | 70-105 | 1605 | Inf | 0 | Inf | 0.990 |  |
| Total | 11-55 | 12994 | Ref |  |  |  |  |
| Total | 56-69 | 29437 | 1.59 | 0.943 | 2.695 | 0.082 |  |
| Total | 70-105 | 27342 | 2.57 | 1.534 | 4.303 | <0.001 |  |
| **SEER disease stage** | **Age of diagnosis** |  |  |  |  |  | 0.862 |
| Localized | 11-55 | 2277 | Ref |  |  |  |  |
| Localized | 56-69 | 6380 | 1.34 | 0.492 | 3.676 | 0.564 |  |
| Localized | 70-105 | 7216 | 2.23 | 0.838 | 5.925 | 0.108 |  |
| Regional | 11-55 | 4123 | 0.79 | 0.227 | 2.727 | 0.706 |  |
| Regional | 56-69 | 9294 | 1.60 | 0.598 | 4.263 | 0.351 |  |
| Regional | 70-105 | 7561 | 3.46 | 1.325 | 9.043 | 0.011 |  |
| Distant | 11-55 | 5433 | 1.29 | 0.388 | 4.261 | 0.681 |  |
| Distant | 56-69 | 10711 | 1.98 | 0.72 | 5.459 | 0.185 |  |
| Distant | 70-105 | 7478 | 3.35 | 1.212 | 9.232 | 0.020 |  |
| Unknown/unstaged | 11-55 | 1161 | 1.22 | 0.237 | 6.316 | 0.809 |  |
| Unknown/unstaged | 56-69 | 3052 | 2.58 | 0.859 | 7.735 | 0.091 |  |
| Unknown/unstaged | 70-105 | 5087 | 2.78 | 0.967 | 7.974 | 0.058 |  |
| Total | 11-55 | 12994 | Ref |  |  |  |  |
| Total | 56-69 | 29437 | 1.69 | 0.998 | 2.855 | 0.051 |  |
| Total | 70-105 | 27342 | 2.90 | 1.729 | 4.878 | <0.001 |  |
| **Surgery performed** | **Age of diagnosis** |  |  |  |  |  | 0.460 |
| Yes | 11-55 | 4471 | Ref |  |  |  |  |
| Yes | 56-69 | 9458 | 1.38 | 0.641 | 2.974 | 0.410 |  |
| Yes | 70-105 | 5299 | 1.70 | 0.733 | 3.942 | 0.216 |  |
| No/Unknown | 11-55 | 8523 | 1.09 | 0.431 | 2.766 | 0.853 |  |
| No/Unknown | 56-69 | 19979 | 2.08 | 0.995 | 4.325 | 0.052 |  |
| No/Unknown | 70-105 | 22043 | 3.55 | 1.744 | 7.206 | 0.001 |  |
| Total | 11-55 | 12994 | Ref |  |  |  |  |
| Total | 56-69 | 29437 | 1.64 | 0.968 | 2.767 | 0.066 |  |
| Total | 70-105 | 27342 | 2.59 | 1.541 | 4.343 | <0.001 |  |
| **Radiotherapy performed** | **Age of diagnosis** |  |  |  |  |  | 0.315 |
| Yes | 11-55 | 7754 | Ref |  |  |  |  |
| Yes | 56-69 | 17434 | 1.23 | 0.633 | 2.402 | 0.539 |  |
| Yes | 70-105 | 14235 | 2.51 | 1.323 | 4.747 | 0.005 |  |
| No/Unknown | 11-55 | 5240 | 0.83 | 0.312 | 2.216 | 0.712 |  |
| No/Unknown | 56-69 | 12003 | 2.07 | 1.064 | 4.011 | 0.032 |  |
| No/Unknown | 70-105 | 13107 | 2.78 | 1.442 | 5.369 | 0.002 |  |
| Total | 11-55 | 12994 | Ref |  |  |  |  |
| Total | 56-69 | 29437 | 1.65 | 0.978 | 2.795 | 0.060 |  |
| Total | 70-105 | 27342 | 2.78 | 1.657 | 4.646 | <0.001 |  |
| **Chemotherapy performed** | **Age of diagnosis** |  |  |  |  |  | 0.583 |
| Yes | 11-55 | 8322 | Ref |  |  |  |  |
| Yes | 56-69 | 17317 | 2.06 | 0.995 | 4.276 | 0.052 |  |
| Yes | 70-105 | 11761 | 3.00 | 1.434 | 6.277 | 0.004 |  |
| No/Unknown | 11-55 | 4672 | 2.04 | 0.807 | 5.146 | 0.132 |  |
| No/Unknown | 56-69 | 12120 | 2.49 | 1.165 | 5.311 | 0.019 |  |
| No/Unknown | 70-105 | 15581 | 4.55 | 2.227 | 9.303 | <0.0001 |  |
| Total | 11-55 | 12994 | Ref |  |  |  |  |
| Total | 56-69 | 29437 | 1.63 | 0.964 | 2.756 | 0.068 |  |
| Total | 70-105 | 27342 | 2.64 | 1.573 | 4.434 | <0.001 |  |
| **SEER disease stage** | **Race** |  |  |  |  |  | 0.746 |
| Localized | Black | 2176 | Ref |  |  |  |  |
| Localized | White | 12939 | 9232461.38 | 0 | Inf | 0.990 |  |
| Localized | Other | 758 | 8164626.62 | 0 | Inf | 0.990 |  |
| Regional | Black | 2745 | 1599021.14 | 0 | Inf | 0.991 |  |
| Regional | White | 17003 | Inf | 0 | Inf | 0.990 |  |
| Regional | Other | 1230 | 9575075.02 | 0 | Inf | 0.990 |  |
| Distant | Black | 3210 | 2094907.18 | 0 | Inf | 0.991 |  |
| Distant | White | 19082 | Inf | 0 | Inf | 0.990 |  |
| Distant | Other | 1330 | Inf | 0 | Inf | 0.990 |  |
| Unknown/unstaged | Black | 1475 | 3505415.17 | 0 | Inf | 0.991 |  |
| Unknown/unstaged | White | 7303 | Inf | 0 | Inf | 0.990 |  |
| Unknown/unstaged | Other | 522 | Inf | 0 | Inf | 0.990 |  |
| Total | Black | 9606 | Ref |  |  |  |  |
| Total | White | 56327 | 7.12 | 2.268 | 22.326 | 0.001 |  |
| Total | Other | 3840 | 8.91 | 2.514 | 31.586 | 0.001 |  |
| **Surgery performed** | **Race** |  |  |  |  |  | 0.517 |
| Yes | Black | 1770 | Ref |  |  |  |  |
| Yes | White | 16579 | 7364532.28 | 0 | Inf | 0.991 |  |
| Yes | Other | 879 | 6485291.82 | 0 | Inf | 0.991 |  |
| No/Unknown | Black | 7836 | 2149277.79 | 0 | Inf | 0.992 |  |
| No/Unknown | White | 39748 | Inf | 0 | Inf | 0.991 |  |
| No/Unknown | Other | 2961 | Inf | 0 | Inf | 0.990 |  |
| Total | Black | 9606 | Ref |  |  |  |  |
| Total | White | 56327 | 7.73 | 2.461 | 24.267 | 0.001 |  |
| Total | Other | 3840 | 9.25 | 2.609 | 32.78 | 0.001 |  |
| **Radiotherapy performed** | **Race** |  |  |  |  |  | 0.260 |
| Yes | Black | 5873 | Ref |  |  |  |  |
| Yes | White | 31220 | 4.35 | 1.374 | 13.793 | 0.012 |  |
| Yes | Other | 2330 | 5.59 | 1.445 | 21.618 | 0.013 |  |
| No/Unknown | Black | 3733 | 0.00 | 0 | Inf | 0.991 |  |
| No/Unknown | White | 25107 | 5.63 | 1.77 | 17.898 | 0.003 |  |
| No/Unknown | Other | 1510 | 7.21 | 1.721 | 30.164 | 0.007 |  |
| Total | Black | 9606 | Ref |  |  |  |  |
| Total | White | 56327 | 6.88 | 2.191 | 21.586 | 0.001 |  |
| Total | Other | 3840 | 8.81 | 2.485 | 31.219 | 0.001 |  |
| **Chemotherapy performed** | **Race** |  |  |  |  |  | 0.708 |
| Yes | Black | 4852 | Ref |  |  |  |  |
| Yes | White | 30505 | 10.67 | 1.483 | 76.776 | 0.019 |  |
| Yes | Other | 2043 | 11.17 | 1.305 | 95.587 | 0.028 |  |
| No/Unknown | Black | 4754 | 3.11 | 0.281 | 34.251 | 0.355 |  |
| No/Unknown | White | 25822 | 16.07 | 2.233 | 115.653 | 0.006 |  |
| No/Unknown | Other | 1797 | 24.10 | 2.964 | 195.925 | 0.003 |  |
| Total | Black | 9606 | Ref |  |  |  |  |
| Total | White | 56327 | 7.01 | 2.233 | 21.985 | 0.001 |  |
| Total | Other | 3840 | 8.90 | 2.512 | 31.554 | 0.001 |  |
| **Surgery performed** | **SEER disease stage** |  |  |  |  |  | 0.485 |
| Yes | Localized | 6533 | Ref |  |  |  |  |
| Yes | Regional | 9422 | 1.34 | 0.714 | 2.519 | 0.362 |  |
| Yes | Distant | 2386 | 1.94 | 0.761 | 4.96 | 0.165 |  |
| Yes | Unknown/unstaged | 887 | 0.62 | 0.082 | 4.643 | 0.640 |  |
| No/Unknown | Localized | 9340 | 2.09 | 1.124 | 3.877 | 0.020 |  |
| No/Unknown | Regional | 11556 | 2.37 | 1.298 | 4.325 | 0.005 |  |
| No/Unknown | Distant | 21236 | 2.00 | 1.092 | 3.676 | 0.025 |  |
| No/Unknown | Unknown/unstaged | 8413 | 2.52 | 1.324 | 4.794 | 0.005 |  |
| Total | Localized | 15873 | Ref |  |  |  |  |
| Total | Regional | 20978 | 1.22 | 0.813 | 1.815 | 0.343 |  |
| Total | Distant | 23622 | 1.09 | 0.689 | 1.716 | 0.718 |  |
| Total | Unknown/unstaged | 9300 | 1.21 | 0.721 | 2.046 | 0.466 |  |
| **Radiotherapy performed** | **SEER disease stage** |  |  |  |  |  | 0.950 |
| Yes | Localized | 8251 | Ref |  |  |  |  |
| Yes | Regional | 14962 | 1.33 | 0.759 | 2.311 | 0.322 |  |
| Yes | Distant | 12007 | 1.25 | 0.659 | 2.375 | 0.494 |  |
| Yes | Unknown/unstaged | 4203 | 1.40 | 0.646 | 3.04 | 0.392 |  |
| No/Unknown | Localized | 7622 | 1.29 | 0.703 | 2.373 | 0.409 |  |
| No/Unknown | Regional | 6016 | 1.60 | 0.804 | 3.171 | 0.181 |  |
| No/Unknown | Distant | 11615 | 1.91 | 0.998 | 3.636 | 0.051 |  |
| No/Unknown | Unknown/unstaged | 5097 | 2.07 | 1.014 | 4.237 | 0.046 |  |
| Total | Localized | 15873 | Ref |  |  |  |  |
| Total | Regional | 20978 | 1.32 | 0.87 | 1.988 | 0.193 |  |
| Total | Distant | 23622 | 1.36 | 0.868 | 2.127 | 0.180 |  |
| Total | Unknown/unstaged | 9300 | 1.51 | 0.907 | 2.519 | 0.113 |  |
| **Chemotherapy performed** | **SEER disease stage** |  |  |  |  |  | 0.305 |
| Yes | Localized | 6458 | Ref |  |  |  |  |
| Yes | Regional | 14075 | 1.17 | 0.647 | 2.105 | 0.608 |  |
| Yes | Distant | 13960 | 1.03 | 0.535 | 1.988 | 0.927 |  |
| Yes | Unknown/unstaged | 2907 | 1.05 | 0.411 | 2.687 | 0.918 |  |
| No/Unknown | Localized | 9415 | 1.17 | 0.632 | 2.18 | 0.612 |  |
| No/Unknown | Regional | 6903 | 1.85 | 0.949 | 3.603 | 0.071 |  |
| No/Unknown | Distant | 9662 | 2.81 | 1.42 | 5.568 | 0.003 |  |
| No/Unknown | Unknown/unstaged | 6393 | 2.09 | 1.053 | 4.15 | 0.035 |  |
| Total | Localized | 15873 | Ref |  |  |  |  |
| Total | Regional | 20978 | 1.47 | 0.964 | 2.227 | 0.074 |  |
| Total | Distant | 23622 | 1.61 | 1.009 | 2.554 | 0.046 |  |
| Total | Unknown/unstaged | 9300 | 1.50 | 0.9 | 2.501 | 0.120 |  |
| **Radiotherapy performed** | **Surgery performed** |  |  |  |  |  | 0.237 |
| Yes | Yes | 10080 | Ref |  |  |  |  |
| Yes | No/Unknown | 29343 | 1.59 | 0.98 | 2.562 | 0.060 |  |
| No/Unknown | Yes | 9148 | 1.08 | 0.61 | 1.92 | 0.787 |  |
| No/Unknown | No/Unknown | 21202 | 2.60 | 1.566 | 4.3 | <0.001 |  |
| Total | Yes | 19228 | Ref |  |  |  |  |
| Total | No/Unknown | 50545 | 1.95 | 1.361 | 2.784 | <0.001 |  |
| **Chemotherapy performed** | **Surgery performed** |  |  |  |  |  | 0.093 |
| Yes | Yes | 10035 | Ref |  |  |  |  |
| Yes | No/Unknown | 27365 | 1.45 | 0.886 | 2.363 | 0.140 |  |
| No/Unknown | Yes | 9193 | 1.13 | 0.638 | 2.007 | 0.672 |  |
| No/Unknown | No/Unknown | 23180 | 2.95 | 1.799 | 4.829 | <0.001 |  |
| Total | Yes | 19228 | Ref |  |  |  |  |
| Total | No/Unknown | 50545 | 1.98 | 1.383 | 2.822 | <0.001 |  |
| **Chemotherapy performed** | **Radiotherapy performed** |  |  |  |  |  | 0.155 |
| Yes | Yes | 29496 | Ref |  |  |  |  |
| Yes | No/Unknown | 7904 | 1.38 | 0.807 | 2.368 | 0.239 |  |
| No/Unknown | Yes | 9927 | 1.94 | 1.221 | 3.072 | 0.005 |  |
| No/Unknown | No/Unknown | 22446 | 1.57 | 1.09 | 2.256 | 0.015 |  |
| Total | Yes | 39423 | Ref |  |  |  |  |
| Total | No/Unknown | 30350 | 1.02 | 0.697 | 1.479 | 0.937 |  |

**Abbreviations:** Ref, Reference; Inf, Infinite; SEER, Surveillance, Epidemiology, and End Results; HR, Hazard Ratio; 95% CI, 95% Confidence Interval

^a^ Likelihood ratio test comparing Cox regression models with and without interaction terms was used as a test for interaction
